# Lifestyle Characteristics and Dietary Habits in Romanian Patients with Periodontitis: A Cross-Sectional Study

**DOI:** 10.3390/healthcare14172676

**Published:** 2026-08-22

**Authors:** Ana Maria Hofer, Andrei Picos, Bogdana Adriana Nasui, Alexandra Dădârlat-Pop, Monica Popa

**Affiliations:** 1 Department of Community Health, “Iuliu Hatieganu” University of Medicine and Pharmacy, 400349 Cluj-Napoca, Romania; 2 Department of Oral Prevention, “Iuliu Hatieganu” University of Medicine and Pharmacy, 400012 Cluj-Napoca, Romania; 3 Department of Cardiology, Heart Institute “N. Stăncioiu”, Calea Moților 19-21, 400001 Cluj-Napoca, Romania; 4 4th Department of Internal Medicine, Faculty of Medicine, “Iuliu Hațieganu” University of Medicine and Pharmacy, 400347 Cluj-Napoca, Romania

**Keywords:** periodontitis, diet quality, nutrition knowledge, dietary habits, nutritional education

## Abstract

**Background/Objectives**: Periodontitis is a multifactorial chronic inflammatory disease influenced by behavioral, metabolic, and lifestyle-related factors. This study aimed to examine the associations of selected dietary habits, body mass index (BMI), physical activity, smoking, and oral hygiene behaviors with clinical periodontal parameters in a clinical sample of adults with periodontitis from northwestern Romania. **Methods**: This cross-sectional study included 204 adults with clinically diagnosed periodontitis. Sociodemographic characteristics, smoking status, physical activity, toothbrushing frequency, tooth mobility, and selected dietary habits were assessed using a structured questionnaire. Periodontal status was clinically evaluated using periodontal probing depth (PPD), clinical attachment loss (CAL), and bleeding on probing (BOP), while BMI was calculated from anthropometric measurements. Descriptive and inferential statistical analyses were performed to evaluate associations between the investigated factors and periodontal parameters. Multivariable linear regression models were constructed separately for PPD, CAL, and BOP, simultaneously including age, sex, BMI, smoking status, physical activity frequency, toothbrushing frequency, tooth mobility, education level, and the assessed dietary habits. **Results**: In the fully adjusted multivariable analyses, fish consumption 1–3 times/week was independently associated with lower PPD, whereas sweets consumption 1–3 times/week was independently associated with higher PPD. Carbonated beverage and sweets consumption 1–3 times/week were independently associated with higher BOP. Although the multivariable model for CAL was statistically significant overall, none of the individual predictors included in the model reached statistical significance. BMI and physical activity frequency were not independently associated with PPD, CAL, or BOP after simultaneous adjustment for the other variables. Age group was significantly associated with CAL category (χ^2^(2) = 13.28, *p* = 0.001, Cramér’s V = 0.255), while educational level was significantly associated with toothbrushing frequency (χ^2^ = 62.26, *p* < 0.001, Cramér’s V = 0.39). **Conclusions**: Selected dietary habits were independently associated with PPD and BOP after adjustment for demographic, anthropometric, lifestyle, and oral hygiene factors, whereas no individual predictor was independently associated with CAL in the fully adjusted model. BMI and physical activity were not independently associated with the periodontal parameters examined. These findings emphasize the multifactorial nature of periodontal status and suggest that specific dietary habits may be associated with different clinical dimensions of periodontal disease. However, causal relationships cannot be established due to the cross-sectional design of the study. Prospective studies using validated dietary assessment methods are needed to confirm these associations.

## 1. Introduction

According to the Romania Health Program-for-Results (PforR), periodontitis affects nearly 66% of the studied population suffering from various forms [1] and is one of the main reasons for tooth loss [2]. A primary risk factor for periodontitis is the accumulation of intraoral dysbiotic microflora at the tooth surface [3]. The host immune response to bacterial colonization involves the breakdown of the periodontal ligament and adjacent bone tissue [4]. Moreover, this immune response is influenced by genetic, environmental, and behavioral factors [5]. The high prevalence of periodontal disease among teenagers, adults and seniors is a public health problem [6]. Age is an important factor in the clinical expression of periodontitis, particularly when substantial periodontal destruction occurs early in life. Periodontitis affecting young individuals may represent a distinct phenotype characterized by rapid attachment and bone loss despite the relatively short duration of exposure to conventional periodontal risk factors. Nibali et al. highlighted that Grade C molar-incisor pattern periodontitis predominantly affects adolescents and young adults and may involve a combination of specific microbial profiles, altered host inflammatory responses, and individual or familial susceptibility. *Aggregatibacter actinomycetemcomitans*, particularly the JP2 clone, has been strongly associated with disease severity and progression, although other microbial species and host-response abnormalities may also contribute. These findings emphasize that age should be considered when interpreting periodontal severity, as similar levels of attachment loss occurring at different ages may reflect different underlying pathogenic mechanisms [7].

Dietary diversity is closely associated with periodontal health status across all age groups [8]. Adequate, balanced nutrition is crucial for preserving the symbiotic relationship between the oral microbiome and periodontal tissues [9]. While numerous macro- and micronutrients reach the gastrointestinal system via saliva, mastication remains a key component of nutrient intake. Effective chewing depends on the integrity of periodontal structures, which are shaped by factors including tobacco use, oral hygiene practices, genetic and epigenetic influences, diet, and overall systemic health [10].

Periodontal diseases are linked to major chronic conditions such as cardiovascular disease, diabetes, stroke, respiratory disease, and rheumatoid arthritis [11,12]. They are associated with increased cardiovascular risk—about 19% overall and up to 44% in older adults—and show a bidirectional relationship with key noncommunicable diseases [6,13,14].

The oral microbiome represents one of the most complex microbial ecosystems in the human body and plays a fundamental role in maintaining oral homeostasis. Rather than being a passive microbial community [15], it actively contributes to host defense by preventing pathogen colonization, regulating immune responses, and preserving the integrity of oral tissues. Disturbances in its composition, known as dysbiosis, are increasingly recognized as key contributors to the development of oral diseases, particularly periodontitis, while also influencing the risk of several systemic conditions. The oral microbiome is highly dynamic and strongly influenced by environmental and behavioral factors. Diet, smoking, alcohol consumption, oral hygiene practices, systemic diseases, medications, and other lifestyle-related exposures continuously shape microbial composition, favoring either a balanced microbial ecosystem or dysbiosis [16]. Consequently, oral microbial communities should be regarded as responsive to modifiable lifestyle factors rather than as static biological entities.

Many recent studies indicate that dietary habits can meaningfully influence periodontal status, with anti-inflammatory and nutrient-dense diets associated with reduced gingival and periodontal inflammation. Interventions such as increased intake of antioxidants (e.g., from green tea), higher fiber, and lower fat or refined carbohydrates show potential to improve periodontal markers and support periodontal health [17,18,19,20].

Diet is one of the principal modulators of the oral microbiome. Fermentable carbohydrates promote the proliferation of acidogenic bacteria and contribute to biofilm dysbiosis, whereas dietary components such as nitrates, antioxidants, and anti-inflammatory compounds may promote a more balanced microbial ecosystem by modifying both microbial composition and host immune responses [21]. Smoking reduces biofilm oxygenation, thereby creating a favorable environment for the proliferation of anaerobic bacteria associated with periodontitis. Alcohol consumption alters salivary composition, impairs host immune defenses, and modifies the relative abundance of bacterial genera such as *Neisseria*, *Prevotella*, and *Streptococcus* [22]. Carbohydrates, vitamins, polyphenols, and other dietary components contribute to maintaining microbial homeostasis or, conversely, promote oral dysbiosis and chronic periodontal inflammation [23]. These findings support the concept that nutrition influences periodontal health not only through direct effects on the host but also by shaping the ecological balance of the oral microbiota.

Beyond influencing microbial composition and growth, nutritional factors may also modulate the virulence of periodontal pathogens. *Porphyromonas gingivalis* produces gingipains (Rgp and Kgp), cysteine proteases involved in nutrient acquisition, biofilm formation, tissue destruction, and immune modulation. Recent evidence indicates that food-derived natural compounds, particularly polyphenols, can inhibit gingipain activity and reduce *P. gingivalis* growth, biofilm formation, and virulence [24]. *Aggregatibacter actinomycetemcomitans* produces leukotoxin A (LtxA), a major virulence factor capable of targeting host immune cells and contributing to immune dysregulation [25]. Recent studies suggest that dietary polyphenols may interfere with periodontal pathogen virulence through downregulation of virulence-associated genes, inhibition of biofilm formation, and modulation of host inflammatory responses [26]. These findings indicate that nutritional factors may influence periodontal dysbiosis not only by altering bacterial abundance, but also by modulating microbial virulence mechanisms.

At present, there are few large epidemiological studies from Romania that directly quantify the association between dietary habits and periodontal disease prevalence or severity [27]. Most existing national research focuses on behavioral elements like smoking and oral hygiene awareness rather than nutrition per se. A cross-sectional study on Romanian adults identified gaps in periodontal awareness and oral hygiene behaviors (e.g., only ~24% floss daily), but did not directly assess diet quality or dietary habits in relation to periodontal outcomes [27].

There are no precise nationwide figures on how many patients specifically access periodontology specialists, but limited public dental service coverage (only ~5% of dental offices are public and cost-free) suggests that access, particularly for specialized periodontal care, may be constrained for many, especially in rural areas. This limited public provision and relatively low routine dental attendance likely restricts access to periodontal evaluation and dietary counseling at the specialist level [28].

To address these gaps, the present study evaluated dietary habits, lifestyle characteristics, anthropometric parameters, and oral hygiene behaviors in Romanian adults with clinically diagnosed periodontitis. By combining questionnaire-based assessment with comprehensive periodontal examination, this study provides evidence from an underrepresented population and contributes to the growing body of literature exploring the interaction between modifiable lifestyle factors and periodontal disease. The objective of this study was to examine the associations of selected dietary habits, BMI, physical activity, smoking, and oral hygiene behaviors with clinical periodontal parameters in a clinical sample.

## 2. Materials and Methods

### 2.1. Study Sample and Data Collection

Because the primary objective of the study was not to determine adherence to a specific dietary habit but to examine the associations of selected dietary habits and other modifiable lifestyle and behavioral factors with periodontal parameters, a study-specific questionnaire was developed instead of adopting an existing dietary scoring instrument. Data were collected using paper-based questionnaires administered to patients in one private dental clinic. The questionnaires were completed on site, at the time patients reviewed and signed the informed consent documents, allowing data collection to be integrated into the routine clinical admission process. The study was conducted with the approval of the participating private clinic and in accordance with the ethical requirements and code of ethics of the “Iuliu Hațieganu” University of Medicine and Pharmacy, Cluj-Napoca (Nr.AVZ208/27 July 2023).

The inclusion criteria were: (1) age ≥ 18 years; (2) diagnosis of periodontitis according to the 2018 AAP/EFP classification, defined by detectable interdental clinical attachment loss (CAL) at ≥2 non-adjacent teeth or buccal/oral CAL ≥ 3 mm with probing depth > 3 mm at ≥2 teeth, where attachment loss could not be attributed to non-periodontal causes; (3) availability of complete periodontal clinical measurements, including probing pocket depth (PPD), CAL, and bleeding on probing (BOP); (4) completion of the study questionnaire regarding demographic, dietary, oral hygiene, and lifestyle characteristics; and (5) provision of informed consent for participation in the study.

Patients were excluded if they submitted incomplete questionnaires, did not undergo the oral health assessment, or presented with systemic chronic diseases (such as diabetes, renal or pulmonary disease, or inflammatory bowel disease), malignancies, cognitive or psychiatric disorders that could influence questionnaire responses, or congenital heart disease. The study was conducted between September 2024 and December 2025. During the predefined recruitment period, a total of 584 patients attended the dental clinic and were assessed for eligibility. Of these, 380 were excluded: 2 participants were younger than 18 years, 43 had no remaining natural teeth, and 335 had no detectable interdental clinical attachment loss. The remaining 204 patients met the eligibility criteria, were enrolled in the study, and completed the questionnaire and clinical periodontal examination. No a priori sample size calculation was performed. The study sample consisted of all eligible participants who met the inclusion criteria and completed the study procedures during the predefined recruitment period. Participation in the study was voluntary, and all eligible participants completed the questionnaire after being informed about the purpose and procedures of the study. Personal data were collected and processed in compliance with applicable data protection regulations. Participants were diagnosed with periodontitis according to the 2018 classification criteria, including interdental clinical attachment loss detectable at ≥2 non-adjacent teeth or buccal/oral clinical attachment loss ≥ 3 mm associated with probing pocket depth > 3 mm at ≥2 teeth, not attributable to non-periodontal causes. Periodontitis stage and grade assignments were established according to the 2018 AAP/EFP classification framework, based on the available clinical periodontal parameters, disease complexity, tooth loss attributable to periodontitis, age-related disease severity, and smoking status [29].

The reporting of this cross-sectional study follows the STROBE (Strengthening the Reporting of Observational Studies in Epidemiology) guidelines (https://www.strobe-statement.org, accessed on 18 August 2026). The completed checklist (Table S1) is available in the Supplementary Materials.

### 2.2. Questionnaire and Variables

We used a self-administered questionnaire. The questionnaire was elaborated/developed based on previous studies and based on international recommendations for a healthy lifestyle and a food frequency questionnaire [30,31]. A pilot questionnaire was tested in a small group of patients to evaluate comprehensibility and completion time before being used in the study population. Moreover, we added some questions regarding periodontal risk factors and the presence of periodontal disease symptoms. The estimated completion time is approximately 20–30 min.

The questionnaire consisted of 28 items, organized into four thematic sections:

Section 1: Demographic and Health Information (Items 1–6).

Collected data on gender, age group, place of residence (urban or rural), and self-reported weight and height in order to calculate the Body Mass Index (BMI). We calculated BMI = Weight (kg)/Height^2^ (m^2^). We divided the sample into underweight, normal weight, overweight, and obese according to World Health Organization (WHO) cut-off points [32]. Data regarding patients’ educational level were also collected.

Section 2: Oral Hygiene Practices, Periodontal Symptoms, and Lifestyle Risk Factors (Items 7–11).

This category encompasses self-reported periodontal symptoms (participants were asked whether they had noticed or perceived mobility of one or more teeth, or gingival bleeding), oral hygiene practices (including frequency of toothbrushing), and lifestyle-related risk factors, specifically alcohol consumption and smoking status.

Section 3: Assessment of Physical Activity Habits and Compliance with WHO Recommendations (Items 12–14).

Section 4: Assessment of Dietary Habits and Food Consumption Frequency (Items 15–28).

The questionnaire consists primarily of closed-ended questions, including single-choice items, where respondents select one option from predefined categories (e.g., sex, age group, education level, frequency of food consumption, smoking status). Dichotomous questions (Yes/No format), used to assess the presence or absence of specific behaviors or conditions (e.g., gingival bleeding, alcohol consumption, breakfast intake). Ordinal frequency-based questions, particularly within the dietary section, assessing consumption patterns (e.g., never, 1–3 times/week, ≥1 time/day). Numeric open-response items, requiring participants to provide quantitative data (e.g., weight in kilograms, height in centimeters, number of days per week of physical activity, minutes per day of exercise). The dietary section was based on a semi-quantitative food frequency approach designed to assess habitual dietary intake rather than short-term dietary consumption.

The primary outcomes were the associations of selected dietary habits, BMI, physical activity, smoking, and oral hygiene behaviors with clinically measured periodontal parameters, including periodontal probing depth (PPD), clinical attachment loss (CAL), and bleeding on probing (BOP). Self-reported oral health indicators, including gingival bleeding and tooth mobility, were analyzed as complementary questionnaire-based variables.

Following questionnaire completion, clinical periodontal parameters were recorded, including periodontal probing depth (PPD), clinical attachment loss (CAL), and bleeding on probing (BOP), providing an objective assessment of periodontal status.

The use of a single examiner was intentionally chosen to minimize inter-examiner variability and enhance measurement consistency, given that periodontal probing measurements may vary according to examiner technique. The examiner used a manual UNC-15 (Hu-Friedy Manufacturing Co., LLC, Chicago, IL, USA) periodontal probe. Probing pocket depth (PPD) was measured from the gingival margin to the base of the periodontal pocket, while clinical attachment loss (CAL) was measured from the cemento-enamel junction (CEJ) to the base of the periodontal pocket. Bleeding on probing (BOP) was recorded following periodontal probing and expressed as the percentage of sites exhibiting bleeding.

To minimize potential measurement bias, all periodontal examinations were performed by a single examiner using a standardized periodontal probe, and the questionnaire was pilot-tested for comprehensibility before administration. All questionnaire responses and clinical measurements were centralized in a unified database and screened for completeness and internal consistency. Questionnaires with substantial missing information and incomplete data were excluded.

### 2.3. Statistical Analysis

All data were recorded in a structured database using Microsoft Excel (Microsoft Corp., Redmond, WA, USA). Statistical analyses were performed using DATAtab (DATAtab Team, 2023; DATAtab e.U., Graz, Austria), an online statistical tool suitable for both descriptive and inferential analysis.

Descriptive statistics were calculated for all variables included in the study. Continuous variables, such as age, body mass index (BMI), periodontal probing depth (PPD), clinical attachment loss (CAL), and bleeding on probing (BOP), were summarized using means, standard deviations, and medians. Categorical variables—including sex, place of residence, smoking status, educational level, dietary habits, and oral hygiene behaviors—were presented as absolute frequencies and percentages.

To assess differences between subgroups (e.g., smokers vs. non-smokers, active vs. inactive lifestyle, high CAL vs. moderate CAL vs. low CAL), inferential statistical tests were applied. The independent samples *t*-test was used to compare the means of continuous variables between two groups when the assumption of normality was met. The normality of data distribution was evaluated using the Shapiro–Wilk test. In cases where the assumption of normality was not satisfied, the non-parametric Mann–Whitney U test was employed.

Associations between categorical variables (e.g., smoking status, dietary habits, oral hygiene habits, and periodontal symptoms such as gingival bleeding or tooth mobility) were evaluated using Cramér’s V coefficient to determine the strength of association.

Multiple linear regression analyses were subsequently performed separately for PPD, CAL, and BOP as dependent variables. Age, sex, BMI, smoking status, physical activity frequency, toothbrushing frequency, tooth mobility, education level, and the assessed dietary habits were entered simultaneously into each model to evaluate their independent associations with the periodontal parameters. Age was categorized as <35 years and ≥35 years. Categorical predictors were entered using the reference categories specified in the corresponding regression tables. Model fit was assessed using R^2^, adjusted R^2^, and the overall F-test. Regression coefficients (B), standardized coefficients (β), 95% confidence intervals (CI), and *p*-values were reported.

Smoking status was assessed as a categorical variable (current smoker, former smoker, non-smoker) and was further analyzed in relation to periodontal indicators and oral hygiene practices.

To further evaluate the potential influence of age on the observed associations, an additional age-stratified analysis was performed. Based on the threshold suggested in the previous periodontal literature, participants were categorized into two age groups: <35 years and ≥35 years. Periodontal parameters and lifestyle-related variables were subsequently analyzed separately within these age strata to explore whether their associations with periodontal severity differed between younger and older participants.

Associations between CAL categories and selected dietary variables were evaluated using Pearson’s Chi-square test. Participants were classified into three mutually exclusive groups according to the highest recorded CAL value: CAL < 3 mm, CAL 3–5 mm, and CAL > 5 mm. For the simplified comparisons presented in the figures, each dietary variable was dichotomized as the selected consumption-frequency category versus all other response categories. The selected category was 1–3 times per week for fish, carbohydrate, red meat, acidophilic dairy product, carbonated beverage, and sweets consumption, whereas fast food consumption was categorized as “never” versus all other responses. Cramér’s V was calculated to quantify the strength of the associations. All tests were two-tailed, and a *p*-value < 0.05 was considered statistically significant.

All statistical tests were two-tailed, and a *p*-value < 0.05 was considered statistically significant. Exact *p*-values were reported when results did not reach statistical significance.

Data visualization, including bar charts, pie charts, and Sankey diagrams, was generated using DATAtab and Microsoft Excel to facilitate interpretation and presentation of findings.

Because of the exploratory nature of the study, analyses primarily focused on identifying potential associations between modifiable lifestyle factors and periodontal disease severity rather than establishing causal relationships.

### 2.4. Ethical Issues

The study was approved by the Bioethics Committee of the University of Medicine and Pharmacy “Iuliu Hațieganu”, Cluj-Napoca, No AVZ208/27.07.2023. The participants were informed about the aim of the study, signed and completed the questionnaire. Participation was voluntary, and all subjects provided written informed consent prior to inclusion in the study and before undergoing the clinical dental examination.

## 3. Results

### 3.1. Participant Characteristics

A total of 204 participants were included in the study. Their demographic, clinical, oral hygiene, and periodontal characteristics are summarized in Table 1. Most participants were aged 30–59 years (62.3%), were female (57.4%), and lived in urban areas (73.0%). The mean BMI was 26.84 ± 4.45 kg/m^2^. Regarding periodontal status, the mean PPD was 5.79 ± 1.40 mm, mean CAL was 3.47 ± 2.34 mm, and mean BOP was 55.56 ± 25.25%. Stage III periodontitis was the predominant stage (69.6%), while Grade B was the most frequent grade (57.4%).

Based on the available periodontal parameters, Stage III represented the predominant severity category, comprising 142 patients (69.6%), followed by Stage I in 29 patients (14.2%), Stage IV in 20 (9.8%), and Stage II in 13 (6.4%). Regarding grading, Grade B was the most frequent category, observed in 117 patients (57.4%), followed by Grade C in 77 (37.7%) and Grade A in 10 (4.9%). When stage and grade were considered together, the most frequent classifications were Stage III Grade B, identified in 78 patients (38.2%), and Stage III Grade C, identified in 57 patients (27.9%). Stage I Grade B was observed in 21 patients (10.3%), while Stage IV Grade B was present in 11 (5.4%). Stage I Grade C, Stage II Grade B, Stage III Grade A, and Stage IV Grade C were each identified in 6–7 patients (2.9–3.4%). Stage IV Grade A was observed in two patients (1.0%), Stage I Grade A in one patient (0.5%), and no participant was classified as Stage II Grade A.

Age-stratified analysis showed differences in the distribution of periodontitis stage and grade between younger and older participants. Among participants aged <35 years (*n* = 64), Stage III was the predominant severity category, identified in 40 patients (62.5%), followed by Stage I in 17 (26.6%) and Stage II in 7 (10.9%); no participant in this age group was classified as Stage IV. Grade B was observed in 36 patients (56.2%) and Grade C in 28 (43.8%). When stage and grade were considered together, Stage III Grade C was the most frequent classification in the younger group (21/64; 32.8%), closely followed by Stage III Grade B (19/64; 29.7%).

Among participants aged ≥35 years (*n* = 140), Stage III was also predominant, occurring in 102 patients (72.9%), while Stage IV was identified in 20 (14.3%), Stage I in 12 (8.6%), and Stage II in 6 (4.3%). Grade B was the predominant grade in this age group (82/140; 58.6%), followed by Grade C (49/140; 35.0%) and Grade A (9/140; 6.4%). The most frequent combined classification was Stage III Grade B (60/140; 42.9%), followed by Stage III Grade C (36/140; 25.7%). Overall, Stage III predominated in both age groups, whereas Stage IV was observed exclusively among participants aged ≥ 35 years.

### 3.2. Oral Hygiene Practices and Periodontal Symptoms

**Figure 1:**
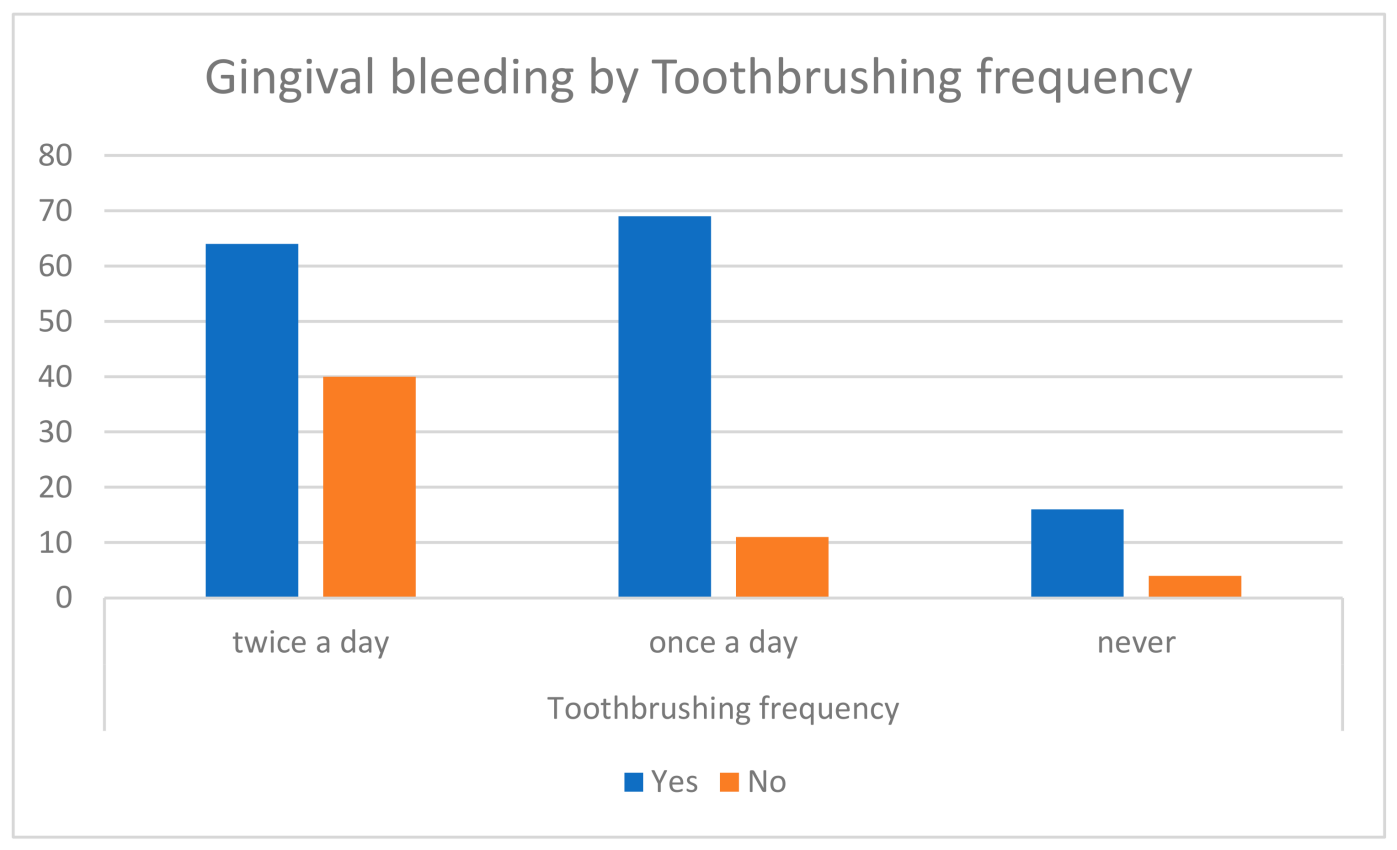
Distribution of self-reported gingival bleeding according to toothbrushing frequency. The association was evaluated using Pearson’s Chi-square test (χ^2^(2) = 14.568, *p* < 0.001).

As illustrated in Figure 1, gingival bleeding was the most frequently reported symptom among participants. Regarding oral hygiene behavior, approximately half of the study population reported brushing twice daily, while a smaller proportion reported brushing once daily or not brushing. Tooth mobility was reported by nearly half of the participants. Overall, the high prevalence of gingival bleeding and tooth mobility, together with suboptimal oral hygiene practices observed in a subset of participants, reflects a high prevalence of periodontal symptoms among the study participants.

### 3.3. Smoking Status and Periodontal Symptoms

**Figure 2:**
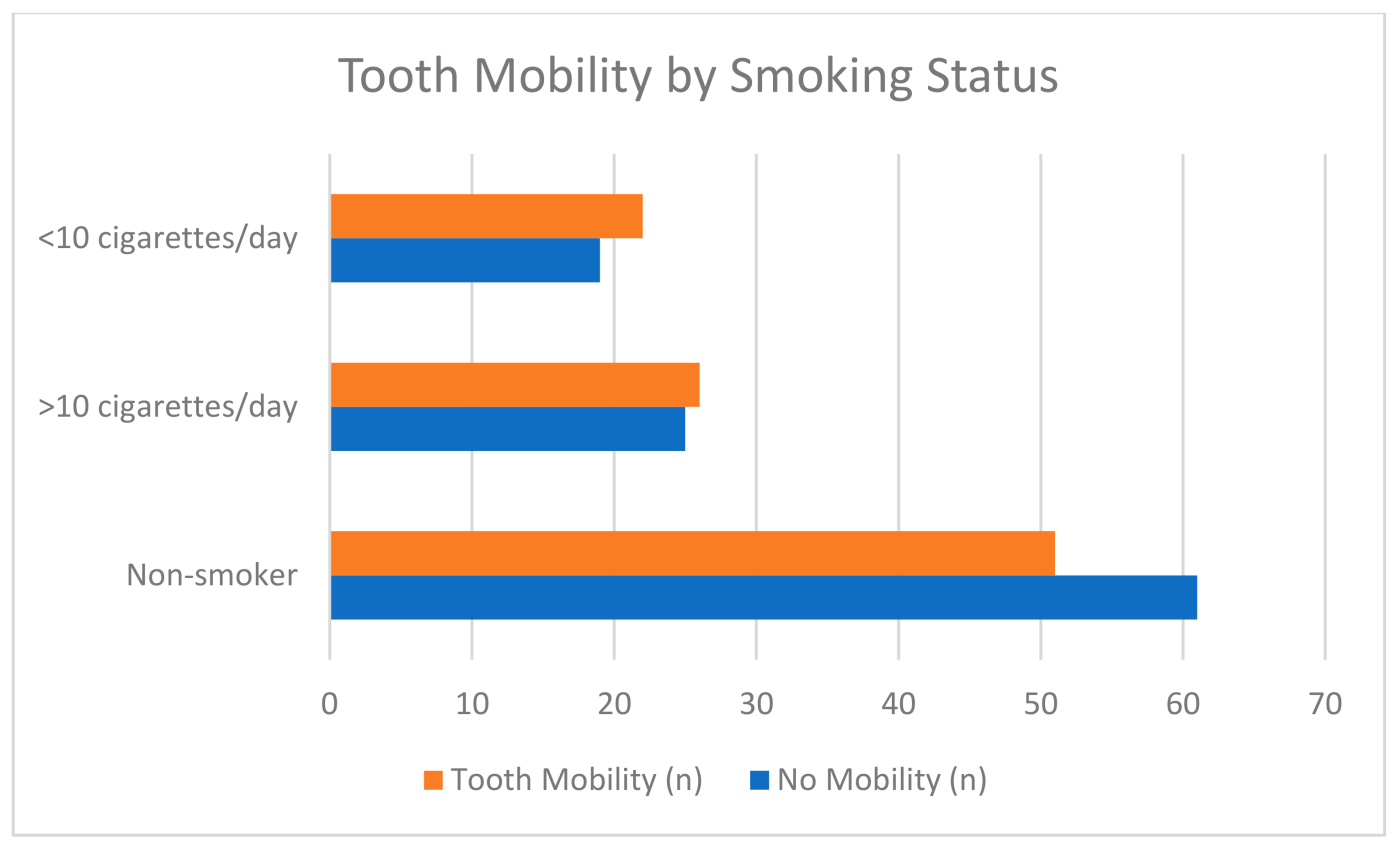
Distribution of self-reported tooth mobility according to smoking status. The association was evaluated using Pearson’s Chi-square test (χ^2^ = 0.95, df = 2, *p* = 0.620).

Self-reported tooth mobility did not differ significantly across smoking-status categories (χ^2^ = 0.95, df = 2, *p* = 0.62). The distribution of tooth mobility was comparable across non-smokers, light smokers, and heavy smokers, as seen in Figure 2.

Furthermore, the strength of association, as measured by Cramér’s V (V = 0.07), indicated a very weak relationship between smoking status and tooth mobility. Nevertheless, a tendency toward lower symptom recognition among smokers compared to non-smokers was observed.

### 3.4. Education and Oral Hygiene Practices

A statistically significant association was found between education level and toothbrushing frequency (χ^2^ = 62.26, df = 4, *p* < 0.001).

Participants with higher education levels were considerably more likely to report brushing their teeth twice daily, whereas individuals with lower educational attainment were more frequently associated with once-daily brushing or the absence of toothbrushing.

Cramér’s V coefficient (V = 0.39) indicated a moderate association between educational level and oral hygiene behavior.

**Figure 3:**
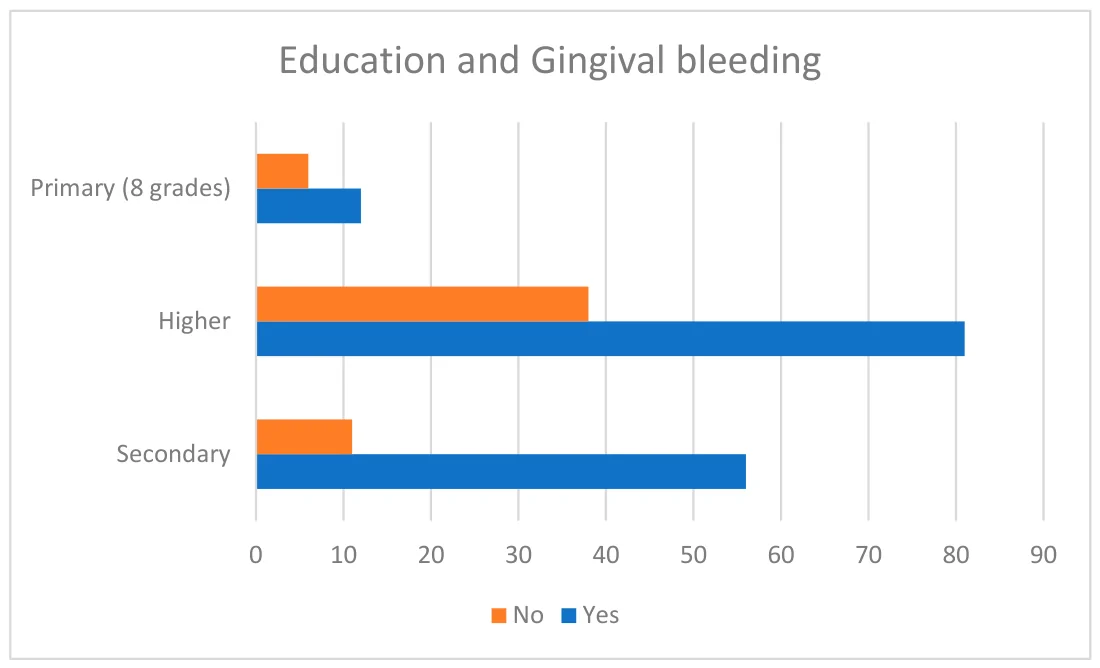
Distribution of self-reported gingival bleeding according to education level. The association was evaluated using Pearson’s Chi-square test (χ^2^ = 5.82, df = 2, *p* = 0.054).

Although the relationship did not reach statistical significance (χ^2^ = 5.82, df = 2, *p* = 0.054), the results approached significance, suggesting that individuals with higher educational attainment may be more likely to recognize gingival bleeding as a periodontal symptom (Figure 3).

Cramér’s V (V = 0.17) indicated a weak association between education level and symptom recognition.

### 3.5. Diet and Periodontal Parameters

**Figure 4:**
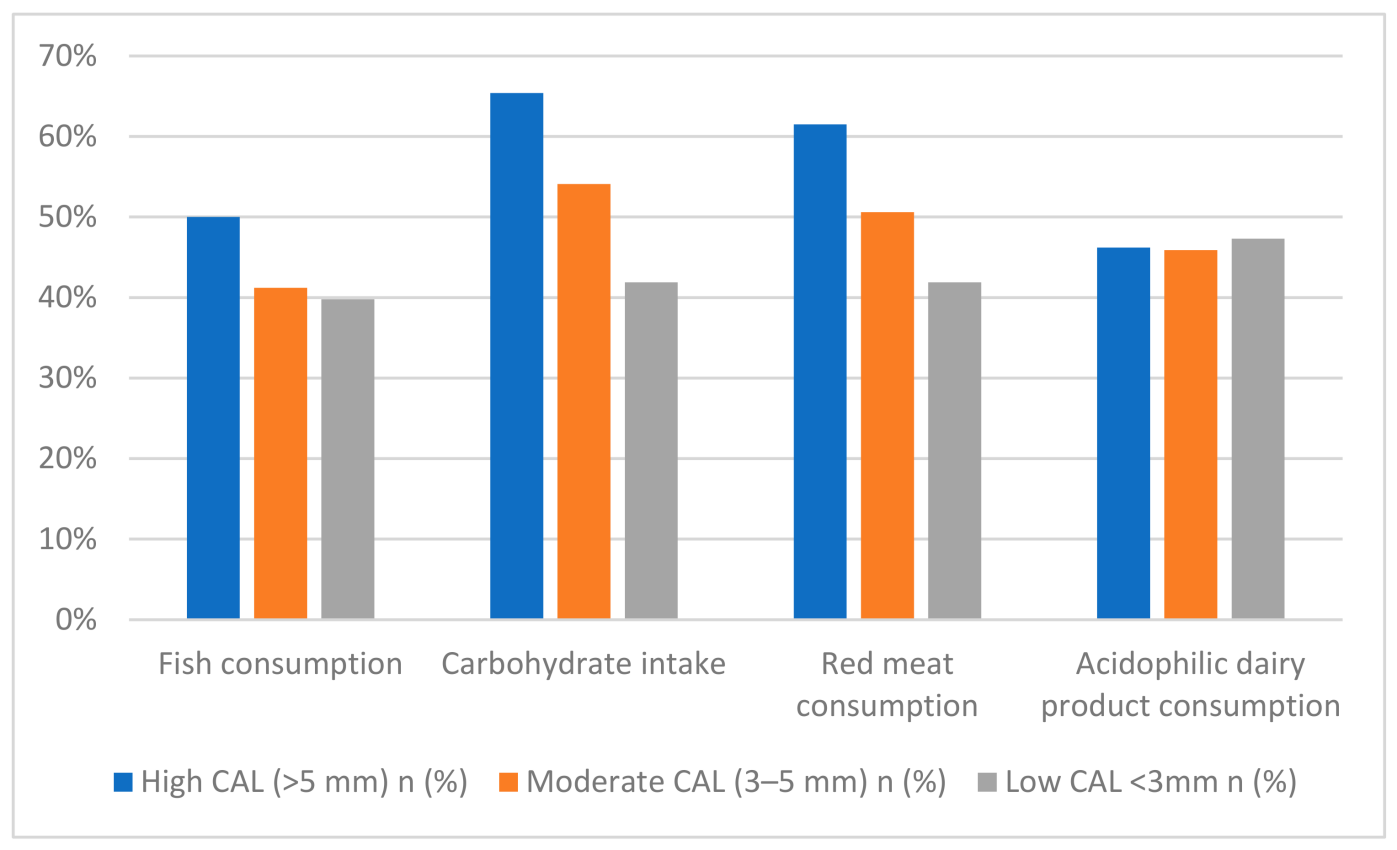
Distribution of the 1–3 times/week consumption category for fish, carbohydrates, red meat, and acidophilic dairy products according to CAL groups (percentages within groups). Associations were evaluated using Pearson’s Chi-square tests: fish, χ^2^(2) = 0.887, *p* = 0.642; carbohydrates, χ^2^(2) = 5.457, *p* = 0.065; red meat, χ^2^(2) = 3.507, *p* = 0.173; and acidophilic dairy products, χ^2^(2) = 0.039, *p* = 0.981.

As seen in Figure 4, participants were classified into three mutually exclusive groups according to the highest recorded clinical attachment loss: CAL < 3 mm (*n* = 93), CAL 3–5 mm (*n* = 85), and CAL > 5 mm (*n* = 26).

The most frequently reported consumption category across the analyzed dietary variables was 1–3 times per week. When the results were expressed as percentages within the three CAL groups—CAL < 3 mm, CAL 3–5 mm, and CAL > 5 mm—some descriptive differences became apparent. Carbohydrate and red meat consumption within the 1–3 times per week category increased progressively across the CAL groups and was most frequently reported among participants with CAL > 5 mm. In contrast, acidophilic dairy product consumption was comparable across the three groups. However, these descriptive differences were not statistically significant and should not be interpreted as evidence of an association between individual dietary variables and CAL severity.

Some dietary behaviors showed descriptive differences across the three CAL groups; however, no statistically significant associations were identified. For fast food consumption, the most frequent response was “never,” reported by 62.4% of participants with CAL < 3 mm, 63.5% of those with CAL 3–5 mm, and 76.9% of those with CAL > 5 mm. Carbonated beverage and sweets consumption were most frequently reported as 1–3 times per week. Carbonated beverage consumption in this category was reported by 35.5%, 50.6%, and 38.5% of participants with CAL < 3 mm, CAL 3–5 mm, and CAL > 5 mm, respectively, while the corresponding percentages for sweets consumption were 59.1%, 56.5%, and 65.4%. Although sweets consumption appeared descriptively more frequent among participants with CAL > 5 mm, no statistically significant associations were observed between these individual dietary variables and CAL categories.

**Figure 5:**
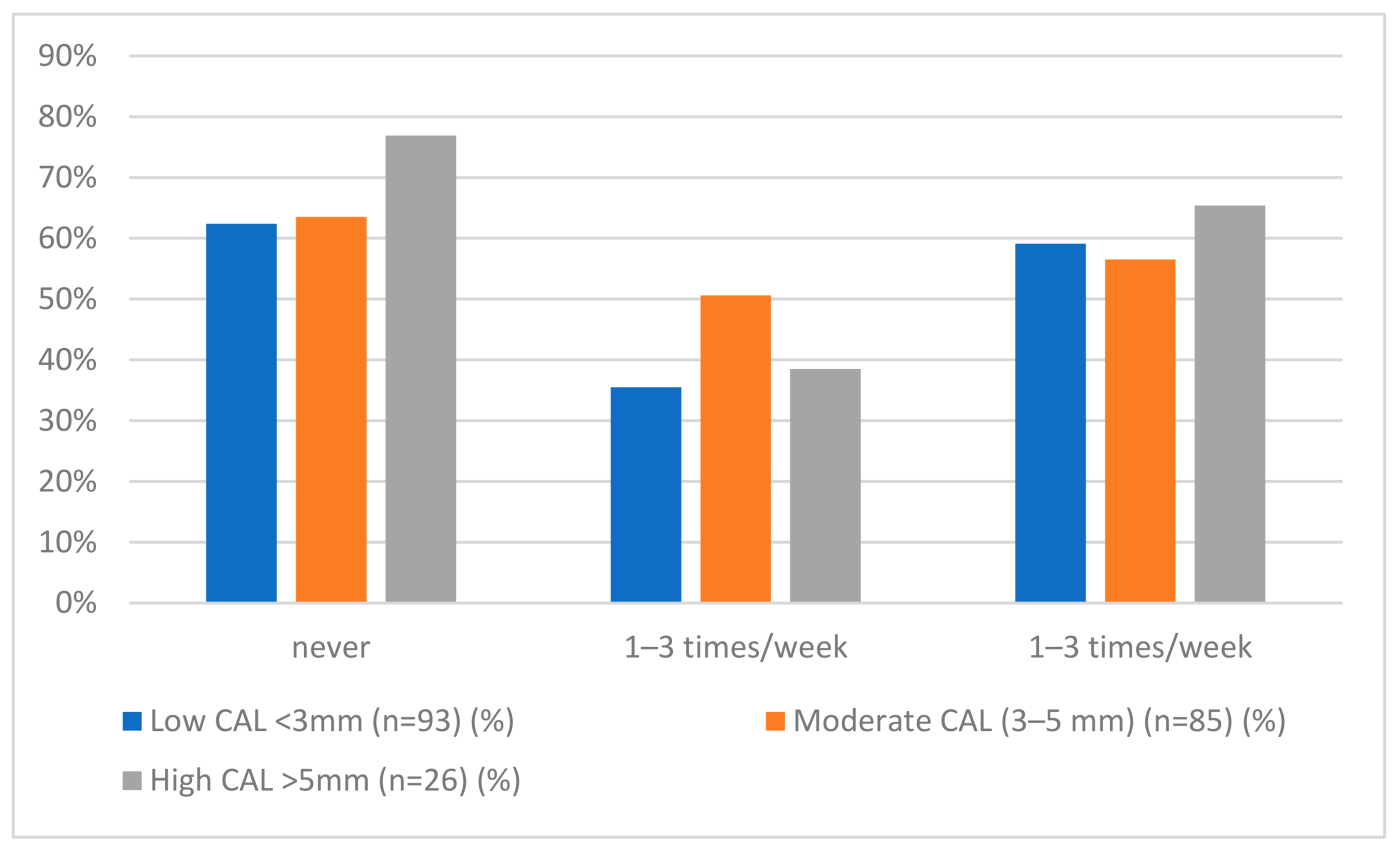
Distribution of the selected consumption-frequency categories for fast food, carbonated beverages, and sweets according to CAL groups (percentages within groups). Associations were evaluated using Pearson’s Chi-square tests: fast food, χ^2^(2) = 1.974, *p* = 0.373; carbonated beverages, χ^2^(2) = 4.322, *p* = 0.115; and sweets, χ^2^(2) = 0.660, *p* = 0.719.

Overall, the analysis of dietary habits across the three Clinical Attachment Loss (CAL) categories revealed predominantly weak and inconsistent patterns (Figure 5). Although carbohydrate and red meat consumption within the 1–3 times/week category appeared to increase across groups and was most frequently reported among participants with CAL > 5 mm, these descriptive differences were not statistically significant and were not consistently observed across the other dietary variables.

Pearson’s Chi-square tests revealed no statistically significant differences among the three CAL groups for the selected dietary categories. Consumption 1–3 times per week was not significantly associated with CAL category for fish (χ^2^(2) = 0.887, *p* = 0.642, Cramér’s V = 0.066), carbohydrates (χ^2^(2) = 5.457, *p* = 0.065, Cramér’s V = 0.164), red meat (χ^2^(2) = 3.507, *p* = 0.173, Cramér’s V = 0.131), acidophilic dairy products (χ^2^(2) = 0.039, *p* = 0.981, Cramér’s V = 0.014), carbonated beverages (χ^2^(2) = 4.322, *p* = 0.115, Cramér’s V = 0.146), or sweets (χ^2^(2) = 0.660, *p* = 0.719, Cramér’s V = 0.057). Similarly, reporting no fast food consumption was not significantly associated with CAL category (χ^2^(2) = 1.974, *p* = 0.373, Cramér’s V = 0.098). Although carbohydrate consumption within the 1–3 times/week category increased descriptively across the CAL groups, the difference did not reach statistical significance.

### 3.6. Multivariable Regression Analysis of Factors Associated with Periodontal Parameters

Multivariable linear regression analyses were performed separately for PPD, CAL, and BOP to assess their associations with demographic, anthropometric, lifestyle, oral hygiene, and dietary factors. Each model simultaneously included age, sex, BMI, smoking status, physical activity frequency, toothbrushing frequency, tooth mobility, education level, and the assessed dietary habits.

The multivariable regression model for PPD was statistically significant overall, indicating that the variables included in the model collectively explained a significant proportion of the variability in PPD (Table 2). After simultaneous adjustment for all covariates, fish consumption 1–3 times/week was significantly associated with lower PPD compared with the other consumption-frequency categories. In contrast, sweets consumption 1–3 times/week was significantly associated with higher PPD. BMI, age, sex, smoking status, physical activity frequency, toothbrushing frequency, tooth mobility, education level, and the remaining dietary variables were not independently associated with PPD. Physical activity showed no independent association with PPD (*p* = 0.993).

The multivariable regression model for CAL was statistically significant overall, suggesting that the predictors considered collectively contributed to explaining variability in attachment loss (Table 3). However, none of the individual variables included in the model reached statistical significance after simultaneous adjustment for the other covariates. Therefore, no independent association with CAL could be demonstrated for BMI, age, sex, smoking status, physical activity frequency, toothbrushing frequency, tooth mobility, education level, or any of the assessed dietary habits. Physical activity frequency was not associated with CAL (*p* = 0.812). These findings indicate that, although the model was significant as a whole, the observed variability in CAL could not be attributed to a single independent predictor within the fully adjusted model.

The multivariable regression model for BOP was statistically significant overall (Table 4). After simultaneous adjustment for all included covariates, carbonated beverage consumption 1–3 times/week was significantly associated with higher BOP values compared with the other consumption-frequency categories. Similarly, sweets consumption 1–3 times/week was independently associated with higher BOP. The positive coefficient for sweets indicated the strongest significant association observed among the predictors retained in the BOP model. In contrast, BMI, age, sex, smoking status, physical activity frequency, toothbrushing frequency, tooth mobility, education level, and the remaining dietary variables were not independently associated with BOP. Physical activity frequency showed no significant independent association with BOP (*p* = 0.937).

### 3.7. Age-Stratified Analysis of Periodontal Severity

To assess the influence of age on periodontal severity, participants were stratified into two groups using a 35-year cut-off: <35 years (*n* = 64) and ≥35 years (*n* = 140). A statistically significant association was observed between age group and CAL category (χ^2^(2) = 13.28, *p* = 0.001, Cramér’s V = 0.255), indicating a small-to-moderate effect size.

Among participants aged < 35 years, 41 (64.1%) presented CAL < 3 mm, 19 (29.7%) presented CAL between 3 and 5 mm, and 4 (6.3%) presented CAL > 5 mm. In comparison, among participants aged ≥ 35 years, 52 (37.1%) presented CAL < 3 mm, 66 (47.1%) presented CAL between 3 and 5 mm, and 22 (15.7%) presented CAL > 5 mm. As shown in the Table 5, greater attachment loss was more frequently observed among participants aged ≥ 35 years, supporting an association between increasing age and greater periodontal severity in the present study population.

Pearson’s Chi-square test showed a statistically significant association between age group and the three CAL categories (χ^2^(2) = 13.28, *p* = 0.0013, Cramér’s V = 0.255). The effect size indicated a small-to-moderate strength of association. Among participants aged < 35 years, 64.1% presented CAL < 3 mm, while only 6.3% presented CAL > 5 mm. In contrast, among participants aged ≥ 35 years, 37.1% presented CAL < 3 mm and 15.7% presented CAL > 5 mm. Higher CAL values were more frequently observed in the ≥35-year age group, indicating greater periodontal attachment loss among older participants.

## 4. Discussion

The present study investigated the associations of selected dietary habits, BMI, physical activity, smoking, oral hygiene behaviors, and sociodemographic characteristics with periodontal status in a cohort of Romanian patients with periodontitis. In the fully adjusted multivariable analyses, distinct associations were observed according to the periodontal outcome assessed. Fish consumption 1–3 times/week was inversely associated with PPD, whereas sweets consumption 1–3 times/week was positively associated with PPD. Carbonated beverage and sweets consumption were positively associated with BOP. In contrast, none of the individual predictors included in the fully adjusted model were significantly associated with CAL. BMI and physical activity were not independently associated with PPD, CAL, or BOP after simultaneous adjustment for the other variables included in the models.

Regarding dietary habits, participants with higher clinical attachment loss (CAL > 5 mm) descriptively reported more frequent carbohydrate and red meat consumption than those with lower CAL values. Sweets consumption also appeared slightly more frequent among participants with CAL > 5 mm. However, these differences were not statistically significant, and the corresponding effect sizes indicated predominantly negligible to weak associations. Therefore, these findings should be interpreted as descriptive patterns rather than evidence of a direct relationship between individual dietary behaviors and periodontal severity.

Although not statistically significant, the observed patterns are consistent with previous evidence suggesting that dietary habits rich in refined carbohydrates and pro-inflammatory foods may contribute to periodontal tissue breakdown through increased systemic inflammation and alterations of the oral microbiome [33,34]. Recent evidence has also emphasized the potential role of dietary composition and diet-derived metabolites in modulating periodontal inflammation and host–microbial interactions [35]. In contrast, healthier dietary habits and nutritional components characterized by higher consumption of fruits, vegetables, fiber, and anti-inflammatory nutrients have been associated with more favorable periodontal outcomes [36].

The lack of significant associations in the present study may also reflect the complex and multifactorial relationship between diet and periodontal health. A recent systematic review and meta-analysis by Aalizadeh et al. reported more favorable periodontal outcomes among individuals with higher adherence to the Mediterranean diet, although the observed association was not statistically significant [37]. Similarly, Wang et al. observed that greater dietary diversity was associated with more favorable periodontal outcomes in a large cohort of older Chinese adults [38]. These findings suggest that comprehensive dietary habits and overall dietary quality may be more relevant to periodontal health than isolated nutrients or food groups evaluated individually.

These findings suggest that comprehensive dietary habits and overall dietary quality may be more relevant to periodontal health than isolated nutrients or food groups evaluated individually. In the present study, dietary intake was assessed using broad food-frequency categories, without information regarding portion size, total caloric intake, nutrient composition, or adherence to validated dietary habits. These methodological differences may partly explain why only weak and inconsistent associations were identified between individual dietary variables and CAL categories.

BMI showed associations with periodontal parameters in the initial analyses; however, these associations were attenuated after simultaneous adjustment for demographic, behavioral, oral hygiene, physical activity, and dietary variables. In the fully adjusted models, BMI was not independently associated with PPD, CAL, or BOP. This attenuation suggests that the relationship between BMI and periodontal status observed in less extensively adjusted analyses may partly reflect the combined influence of other lifestyle and behavioral characteristics. Nevertheless, the direction of the association between BMI and CAL remained positive, although it did not reach statistical significance in the fully adjusted model. These findings do not exclude a potential relationship between excess body weight and periodontal disease but indicate that BMI was not an independent predictor of the periodontal parameters when the broader set of covariates was considered simultaneously. These findings support previous evidence indicating that obesity and excess adipose tissue may contribute to a chronic low-grade inflammatory state that can exacerbate periodontal inflammation and tissue destruction [2,4]. Adipose tissue-derived cytokines and inflammatory mediators have been proposed as possible biological mechanisms linking obesity and periodontitis [36]. Therefore, the association between BMI and periodontal parameters represents a more consistent finding in the present sample than the descriptive differences observed for individual dietary variables.

The associations observed for PPD and BOP further support the potential relationship between increased BMI and periodontal inflammatory burden. PPD reflects the depth of the periodontal pocket and is an important clinical indicator of periodontal disease severity, whereas BOP primarily reflects the presence of gingival inflammation. The multivariable analyses further clarified the relationship between BMI and periodontal status after accounting for age, sex, and smoking status as relevant potential confounders. The association between BMI and PPD was attenuated after adjustment and no longer reached statistical significance, suggesting that part of the unadjusted relationship may be influenced by demographic and behavioral factors, particularly smoking. BMI was also positively associated with BOP in the adjusted analysis. However, the overall model for BOP did not reach statistical significance and explained only a limited proportion of the variability in this parameter. Therefore, this finding should be interpreted in the context of the non-significant overall model and requires confirmation in larger studies.

However, the cross-sectional design does not allow the direction of the relationship between BMI and periodontal status to be established. Increased BMI may contribute to systemic and periodontal inflammation, but periodontal disease may also coexist with other metabolic and behavioral factors that were not comprehensively evaluated. Consequently, BMI should be interpreted as an associated factor rather than a causal determinant of periodontal destruction.

Physical activity frequency was not independently associated with PPD, CAL, or BOP in any of the fully adjusted models. Although regular physical activity has recognized systemic health benefits, its direct relationship with periodontal status remains less clearly established. In the present study, the absence of an association may also reflect the relatively simple assessment of physical activity frequency, which did not comprehensively capture exercise intensity, duration, or long-term activity patterns. Similar findings have been reported previously, as the relationship between physical activity and periodontal status appears less consistent than that of nutritional status and body composition [28,34,39,40]. While regular exercise has recognized systemic health benefits, its direct influence on periodontal health remains less clearly established. In the present study, the lack of association may also be related to the categorical assessment of physical activity, which did not capture exercise intensity, duration, or long-term activity patterns [39,40].

Age represents an important modifier of periodontal disease expression, as younger individuals may present distinct microbial and host-response characteristics, particularly in Grade C molar-incisor pattern periodontitis, as highlighted by Nibali et al. Age may also modify the relationship between lifestyle characteristics and periodontal severity. Considering the broad age range of the study population, an additional analysis was therefore performed after stratifying participants into those aged <35 years and ≥35 years. This distinction is relevant because periodontal destruction in younger individuals may be influenced to a greater extent by individual susceptibility and specific microbial and virulence-related factors, whereas in older individuals the cumulative exposure to modifiable lifestyle and metabolic factors may become increasingly important. The age-stratified findings should nevertheless be interpreted cautiously, particularly because subdivision of the study population reduces the number of participants available for individual comparisons. Although age-stratified analyses were performed to explore potential differences between younger and older participants, the resulting subgroup sizes were smaller and may have limited the statistical power to detect modest associations between lifestyle factors and periodontal severity.

The present study also highlighted the role of educational attainment in shaping oral health behaviors. Participants with higher educational levels were more likely to report toothbrushing twice daily, suggesting that education and health awareness may influence preventive oral hygiene practices. This finding is consistent with previous studies showing that socioeconomic and educational factors can affect both preventive behaviors and periodontal outcomes [27].

The present findings also agree with a recent cross-sectional study conducted in a northwestern Romanian population, which reported substantial deficiencies in the recognition of early periodontal symptoms and inconsistent oral hygiene behaviors [27]. Although more than half of the participants in the present study reported brushing their teeth twice daily, a considerable proportion brushed only once daily or reported inadequate oral hygiene practices. The significant association between educational level and toothbrushing frequency suggests that educational interventions may contribute to improving oral health awareness and preventive behaviors.

Nevertheless, educational attainment should not be interpreted as a direct determinant of periodontal status. Toothbrushing frequency was self-reported and did not provide information regarding brushing technique, duration, interdental cleaning, or long-term plaque control. Therefore, the findings primarily demonstrate an association between education and reported oral hygiene behavior rather than a direct relationship between education and periodontal destruction.

Several other analyzed factors did not show statistically significant associations with periodontal outcomes. Smoking is a well-established adverse modifier of periodontal health, and recent studies have associated cigarette smoking with greater probing depths, increased clinical attachment loss, alveolar bone loss, and more advanced stages of periodontitis [41,42,43]. However, in the present study, smoking status was not significantly associated with self-reported tooth mobility. This finding should not be interpreted as evidence of an absence of detrimental periodontal effects of smoking. Rather, the analysis focused on perceived tooth mobility, a specific and self-reported symptom that may not adequately reflect the extent of smoking-related periodontal tissue destruction and may also be influenced by attachment and bone loss, occlusal factors, and individual perception. Similarly, although some differences were observed between educational groups regarding self-reported gingival bleeding, the association did not reach statistical significance. These findings should not be interpreted as evidence that smoking or educational factors are irrelevant to periodontal health, but rather that the selected relationships could not be demonstrated in the present sample.

Several limitations of this study should be acknowledged. First, the cross-sectional design does not allow causal relationships to be established between dietary habits, lifestyle characteristics, and periodontal outcomes. Consequently, the identified associations and descriptive patterns should be interpreted as exploratory rather than causal.

Second, dietary information and lifestyle-related variables were collected using self-reported questionnaires, which may be subject to recall bias and social desirability bias. Participants may have underreported unhealthy dietary behaviors or overreported favorable health practices.

Third, the study population was recruited from a single private dental clinic in north-western Romania, which may limit the generalizability of the findings to the broader Romanian population, particularly to individuals with limited access to dental care. Additionally, no a priori sample size calculation was performed, which may have limited the statistical power to detect modest associations, particularly in subgroup analyses.

Additionally, although clinical periodontal parameters were objectively recorded, dietary intake was assessed through food-frequency categories rather than through detailed quantitative nutritional analysis. Therefore, the exact intake of specific nutrients, calories, portion sizes, or dietary components could not be determined. This approach may have limited the ability to identify associations between overall dietary quality and periodontal status.

Toothbrushing frequency was self-reported and did not provide information regarding brushing technique, duration, interdental cleaning, mouthwash use, or long-term plaque control. In particular, the use of mouthwash as an adjunctive oral hygiene measure was not assessed, which may have limited the comprehensive characterization of participants’ oral hygiene practices. Therefore, the findings primarily demonstrate an association between education and reported toothbrushing behavior rather than a direct relationship between education and periodontal destruction.

Finally, potential confounding factors such as socioeconomic status, income level, stress, and other health-related behaviors may have influenced both dietary habits and periodontal status and were not comprehensively evaluated in the present analysis.

Although the study was conducted in a single Romanian clinical setting, it contributes data from an Eastern European population that remains underrepresented in periodontal nutrition research. The findings are not intended to establish population-specific causal relationships, but rather to evaluate whether associations previously reported in other settings were also observable in this clinical sample.

Overall, the findings support the multifactorial etiology of periodontitis and highlight the complex interplay among dietary habits, obesity-related factors, educational characteristics, and oral hygiene behaviors. Although individual dietary variables were not significantly associated with CAL categories, BMI was consistently associated with the major periodontal parameters, while educational attainment was associated with toothbrushing frequency. Further prospective and interventional studies using validated dietary assessment methods are required to determine whether overall dietary habits, dietary modification, and nutritional counseling may contribute to the prevention and management of periodontal disease.

## 5. Conclusions

Within the limitations of this cross-sectional study, the fully adjusted multivariable analyses identified specific dietary associations with periodontal parameters. Fish consumption 1–3 times/week was associated with lower PPD, whereas sweets consumption was associated with higher PPD. Carbonated beverage and sweets consumption were also associated with higher BOP values. In contrast, none of the individual predictors examined were independently associated with CAL in the fully adjusted model. BMI and physical activity frequency were not independently associated with PPD, CAL, or BOP after simultaneous adjustment for demographic, behavioral, oral hygiene, and dietary factors. Educational level was significantly associated with toothbrushing frequency, further supporting the relevance of socioeconomic and behavioral characteristics to periodontal health.

These findings suggest that selected dietary and behavioral factors may be associated with different dimensions of periodontal status. However, causal relationships cannot be established due to the cross-sectional design of the study. Prospective studies using validated dietary assessment instruments are required to confirm these associations and clarify their potential clinical relevance.

## Figures and Tables

**Table 1:** Demographic, clinical, oral hygiene, and periodontal characteristics of the study participants (*n* = 204).

Characteristic	Group/Category	*n* (%) or Mean ± SD
Age Group	<35 years	64 (31.4%)
	≥35 years	140 (68.6%)
Gender	Male	117 (57.4%)
	Female	87 (42.6%)
Residence	Urban	149 (73.0%)
	Rural	55 (27.0%)
BMI (kg/m^2^)	-	26.84 ± 4.45
PPD (mm)	-	5.79 ± 1.40
CAL (mm)	-	3.47 ± 2.34
BOP (%)	-	55.56 ± 25.25
Self-reported gingival bleeding	Yes	149 (73.0%)
	No	55 (27.0%)
Toothbrushing frequency	Twice daily	104 (51.0%)
	Once daily	80 (39.2%)
	Never	20 (9.8%)
Self-reported tooth mobility	Yes	99 (48.5%)
	No	105 (51.5%)
Periodontitis stage	Stage I	29 (14.2%)
	Stage II	13 (6.4%)
	Stage III	142 (69.6%)
	Stage IV	20 (9.8%)
Periodontitis grade	Grade A	10 (4.9%)
	Grade B	117 (57.4%)
	Grade C	77 (37.7%)

**Note:** *Data are presented as n (%) for categorical variables and mean ± standard deviation (SD) for continuous variables. BMI, body mass index; PPD, periodontal probing depth; CAL, clinical attachment loss; BOP, bleeding on probing*.

**Table 2:** Multivariable linear regression analysis of factors associated with periodontal probing depth (PPD).

Model	Unstandardized Coefficients		Standardized Coefficients	t	Sig.	95% CI	
	B	Std. Error	Beta			Lower	Upper
**PPD**	4.179	0.665	—	6.279	<0.001	2.866	5.491
**BMI**	0.034	0.025	0.109	1.380	0.169	−0.015	0.083
**Age ≥ 35 years (vs. <35 years)**	0.131	0.218	0.044	0.601	0.549	−0.299	0.561
**Male (vs. female)**	−0.042	0.210	−0.015	−0.202	0.840	−0.456	0.371
**Smoker (vs. non-smoker)**	0.340	0.197	0.121	1.726	0.086	−0.049	0.728
**Physical activity frequency**	0.001	0.072	0.001	0.008	0.993	−0.141	0.142
**Toothbrushing once daily (vs. twice daily)**	−0.117	0.224	−0.041	−0.522	0.602	−0.559	0.325
**Toothbrushing never (vs. twice daily)**	0.369	0.392	0.079	0.940	0.348	−0.405	1.143
**Tooth mobility: Yes (vs. No)**	−0.034	0.208	−0.012	−0.162	0.871	−0.444	0.377
**Secondary education (vs. higher education)**	0.223	0.231	0.075	0.967	0.335	−0.232	0.679
**Primary education (vs. higher education)**	−0.056	0.395	−0.011	−0.142	0.888	−0.834	0.723
**Fish, 1–3 times/week**	−0.431	0.198	−0.152	−2.176	0.031	−0.821	−0.040
**Carbohydrates, 1–3 times/week**	−0.188	0.198	−0.067	−0.951	0.343	−0.578	0.202
**Red meat, 1–3 times/week**	0.282	0.194	0.101	1.455	0.147	−0.101	0.666
**Acidophilic dairy products, 1–3 times/week**	−0.282	0.195	−0.101	−1.446	0.150	−0.667	0.103
**Fast food: Never**	0.425	0.215	0.146	1.977	0.049	0.001	0.849
**Carbonated beverages, 1–3 times/week**	0.104	0.202	0.037	0.516	0.606	−0.295	0.503
**Sweets, 1–3 times/week**	0.638	0.207	0.225	3.077	0.002	0.229	1.047

B, unstandardized regression coefficient; Beta, standardized regression coefficient; CI, confidence interval. Reference categories: age < 35 years, female sex, non-smoker, toothbrushing twice daily, no tooth mobility, and higher education. Physical activity frequency was coded as days/week (no activity = 0; a few minutes/day and 1 day/week = 1; 2, 3, 4, and 7 days/week retained their corresponding values). Dietary variables were coded as the selected consumption-frequency category versus all other response categories; fast food “Never” was compared with all other frequencies. Model fit: R^2^ = 0.188, adjusted R^2^ = 0.114, F(17,186) = 2.531, *p* = 0.001.

**Table 3:** Multivariable linear regression analysis of factors associated with clinical attachment loss (CAL).

Model	Unstandardized Coefficients		Standardized Coefficients	t	Sig.	95% CI	
	B	Std. Error	Beta			Lower	Upper
**CAL**	−0.235	1.133	—	−0.207	0.836	−2.470	2.000
**BMI**	0.074	0.042	0.140	1.754	0.081	−0.009	0.157
**Age ≥ 35 years (vs. <35 years)**	0.672	0.371	0.134	1.811	0.072	−0.060	1.405
**Male (vs. female)**	0.504	0.357	0.107	1.412	0.160	−0.200	1.208
**Smoker (vs. non-smoker)**	0.015	0.335	0.003	0.044	0.965	−0.646	0.676
**Physical activity frequency**	−0.029	0.122	−0.017	−0.239	0.812	−0.270	0.212
**Toothbrushing once daily (vs. twice daily)**	0.409	0.381	0.086	1.074	0.284	−0.343	1.162
**Toothbrushing never (vs. twice daily)**	−0.356	0.668	−0.045	−0.533	0.595	−1.674	0.962
**Tooth mobility: Yes (vs. No)**	0.399	0.354	0.085	1.126	0.262	−0.300	1.097
**Secondary education (vs. higher education)**	0.487	0.393	0.098	1.238	0.217	−0.289	1.262
**Primary education (vs. higher education)**	−0.177	0.672	−0.021	−0.263	0.793	−1.502	1.148
**Fish, 1–3 times/week**	0.194	0.337	0.041	0.576	0.565	−0.471	0.859
**Carbohydrates, 1–3 times/week**	0.438	0.337	0.094	1.301	0.195	−0.226	1.102
**Red meat, 1–3 times/week**	0.529	0.331	0.113	1.599	0.112	−0.124	1.181
**Acidophilic dairy products, 1–3 times/week**	−0.176	0.332	−0.038	−0.530	0.597	−0.831	0.479
**Fast food: Never**	0.043	0.366	0.009	0.119	0.906	−0.678	0.765
**Carbonated beverages, 1–3 times/week**	0.093	0.344	0.020	0.271	0.787	−0.586	0.772
**Sweets, 1–3 times/week**	0.135	0.353	0.028	0.381	0.703	−0.562	0.831

B, unstandardized regression coefficient; Beta, standardized regression coefficient; CI, confidence interval. Reference categories: age < 35 years, female sex, non-smoker, toothbrushing twice daily, no tooth mobility, and higher education. Physical activity frequency was coded as days/week (no activity = 0; a few minutes/day and 1 day/week = 1; 2, 3, 4, and 7 days/week retained their corresponding values). Dietary variables were coded as the selected consumption-frequency category versus all other response categories; fast food “Never” was compared with all other frequencies. Model fit: R^2^ = 0.188, adjusted R^2^ = 0.114, F(17,186) = 2.531, *p* = 0.001.

**Table 4:** Multivariable linear regression analysis of factors associated with bleeding on probing (BOP).

Model	Unstandardized Coefficients		Standardized Coefficients	t	Sig.	95% CI	
	B	Std. Error	Beta			Lower	Upper
**BOP**	27.891	12.031	—	2.318	**0.022**	4.157	51.626
**BMI**	0.491	0.448	0.086	1.097	0.274	−0.392	1.374
**Age ≥ 35 years (vs. <35 years)**	1.621	3.944	0.030	0.411	0.681	−6.159	9.401
**Male (vs. female)**	−3.247	3.790	−0.064	−0.857	0.393	−10.723	4.229
**Smoker (vs. non-smoker)**	3.310	3.557	0.065	0.930	0.353	−3.708	10.328
**Physical activity frequency**	0.102	1.296	0.006	0.079	0.937	−2.455	2.659
**Toothbrushing once daily (vs. twice daily)**	5.868	4.048	0.114	1.450	0.149	−2.118	13.855
**Toothbrushing never (vs. twice daily)**	7.749	7.094	0.092	1.092	0.276	−6.246	21.745
**Tooth mobility: Yes (vs. No)**	−0.055	3.760	−0.001	−0.015	0.988	−7.473	7.363
**Secondary education (vs. higher education)**	2.570	4.174	0.048	0.616	0.539	−5.664	10.805
**Primary education (vs. higher education)**	13.144	7.134	0.148	1.843	0.067	−0.929	27.217
**Fish, 1–3 times/week**	−0.582	3.579	−0.011	−0.163	0.871	−7.643	6.478
**Carbohydrates, 1–3 times/week**	−5.080	3.574	−0.101	−1.421	0.157	−12.130	1.971
**Red meat, 1–3 times/week**	1.005	3.511	0.020	0.286	0.775	−5.922	7.931
**Acidophilic dairy products, 1–3 times/week**	−3.459	3.526	−0.069	−0.981	0.328	−10.414	3.497
**Fast food: Never**	1.394	3.883	0.026	0.359	0.720	−6.268	9.055
**Carbonated beverages, 1–3 times/week**	8.915	3.655	0.175	2.439	**0.016**	1.704	16.126
**Sweets, 1–3 times/week**	12.458	3.750	0.243	3.322	**0.001**	5.060	19.856

B, unstandardized regression coefficient; Beta, standardized regression coefficient; CI, confidence interval. Reference categories: age < 35 years, female sex, non-smoker, toothbrushing twice daily, no tooth mobility, and higher education. Physical activity frequency was coded as days/week (no activity = 0; a few minutes/day and 1 day/week = 1; 2, 3, 4, and 7 days/week retained their corresponding values). Dietary variables were coded as the selected consumption-frequency category versus all other response categories; fast food “Never” was compared with all other frequencies. Model fit: R^2^ = 0.188, adjusted R^2^ = 0.114, F(17,186) = 2.531, *p* = 0.001.

**Table 5:** Distribution of Clinical Attachment Loss (CAL) categories according to age group (<35 years and ≥35 years).

Age Group	CAL < 3 mm, *n* (%)	CAL 3–5 mm, *n* (%)	CAL > 5 mm, *n* (%)
**<35 years**	41 (64.1%)	19 (29.7%)	4 (6.3%)
**≥35 years**	52 (37.1%)	66 (47.1%)	22 (15.7%)
**Total**	93	85	26

## Data Availability

The data presented in this study are available on request from the corresponding author due to the presence of personal data that are not relevant to the study.

## Supplementary Materials

The following supporting information can be downloaded at: https://www.mdpi.com/article/10.3390/healthcare14172676/s1, Table S1: STROBE Statement—Checklist of items included in the cross-sectional study.

## Abbreviations

The following abbreviations are used in this manuscript:

PforR	Program-for-Results
BMI	Body Mass Index
WHO	World Health Organization
PPD	Probing Pocket Depth
CAL	Clinical Attachment Loss
BOP	Bleeding on Probing
SD	Standard Deviation
CI	Confidence Interval
