# Supplementary material for: Lifestyle Characteristics and Dietary Habits in Romanian Patients with Periodontitis: A Cross-Sectional Study
Source: Healthcare (Basel). 2026 Aug 22;14(17):2676. doi: 10.3390/healthcare14172676 (PMC13565102; doi:10.3390/healthcare14172676)
Supplement: Supplementary file 1 [file healthcare-14-02676-s001.zip › healthcare-4452415-supplementary.pdf]

Table S1. STROBE Statement—Checklist of items included in the ***cross-sectional study***

	Item No	Recommendation	Page
**Title and abstract**	1	(*a*) Indicate the study's design with a commonly used term in the title or the abstract: cross-sectional study	1
		(*b*) Provide in the abstract an informative and balanced summary of what was done and what was found: The abstract provides a structured summary of the study background, methods, main results, and conclusions.	1-2
**Introduction**			
Background/rationale	2	Explain the scientific background and rationale for the investigation being reported: The Introduction describes the scientific background and rationale for investigating the associations of dietary and lifestyle-related factors with periodontal status, with particular emphasis on the limited evidence available from Romanian populations.	2-4
Objectives	3	State specific objectives, including any prespecified hypotheses: the Introduction: to examine the associations of selected dietary habits, BMI, physical activity, smoking, and oral hygiene behaviors with clinical periodontal parameters in a clinical sample.	4
**Methods**			
Study design	4	Present key elements of study design early in the paper: cross-sectional design	1,4
Setting	5	Describe the setting, locations, and relevant dates, including periods of recruitment, exposure, follow-up, and data collection: The study setting, geographic location, and recruitment/data collection period are described in the Materials and Methods section. Participants were recruited from a private dental clinic in north-western Romania between September 2024 and December 2025.	4
Participants	6	(*a*) Give the eligibility criteria, and the sources and methods of selection of participants: Eligibility criteria, exclusion criteria, and the source and method of participant selection are described in the Study Sample and Data Collection section.	4-5
Variables	7	Clearly define all outcomes, exposures, predictors, potential confounders, and effect modifiers. Give diagnostic criteria, if applicable: The main study variables are defined in the Materials and Methods section, including clinically measured periodontal parameters (PPD, CAL, and BOP), BMI, dietary habits, physical activity, smoking status, oral hygiene behaviors, and potential confounders used in the adjusted analyses.	5-6
Data sources/ measurement	8*	For each variable of interest, give sources of data and details of methods of assessment (measurement). Describe comparability of assessment methods if there is more than one group: Data sources and measurement procedures are described for questionnaire-derived variables, anthropometric variables, and clinical periodontal parameters. PPD, CAL, and BOP were assessed clinically using standardized periodontal examination procedures.	5-6
Bias	9	Describe any efforts to address potential sources of bias: Potential sources of measurement bias were addressed by using standardized periodontal examination procedures and a single examiner, and the study-specific questionnaire was pilot-tested for comprehensibility before administration.	4-5
Study size	10	Explain how the study size was arrived at: During the predefined recruitment	5

		period, 584 patients attended the dental clinic. Of these, 204 met the study inclusion criteria and were enrolled. No a priori sample size calculation was performed; the final sample comprised all eligible participants who completed the required study procedures during the recruitment period.	
Quantitative variables	11	Explain how quantitative variables were handled in the analyses. If applicable, describe which groupings were chosen and why: The handling of quantitative variables is described in the Statistical Analysis section. Continuous variables were analyzed as appropriate, and the rationale and boundaries for categorized variables, including CAL and age groups (<35 and ≥35 years), are reported.	5-6
Statistical methods	12	(*a*) Describe all statistical methods, including those used to control for confounding: Multiple linear regression analyses were performed separately for PPD, CAL, and BOP. Age, sex, BMI, smoking status, physical activity frequency, toothbrushing frequency, tooth mobility, education level, and the assessed dietary habits were entered simultaneously into each model to evaluate their independent associations with periodontal parameters.	6-7
		(*b*) Describe any methods used to examine subgroups and interactions: Age-stratified analyses were performed using two predefined age groups (<35 and ≥35 years) to explore differences in periodontal and lifestyle-related variables according to age.	6-7
		(*c*) Explain how missing data were addressed: Questionnaires with substantial missing or incomplete information were excluded. The final analytical dataset contained complete data for the variables included in the reported analyses.	6
		(*d*) If applicable, describe analytical methods taking account of sampling strategy: no complex sampling strategy was used.	-
		(*e*) Describe any sensitivity analyses: no sensitivity analyses were performed.	-
**Results**			
Participants	13*	(a) Report numbers of individuals at each stage of study—eg numbers potentially eligible, examined for eligibility, confirmed eligible, included in the study, completing follow-up, and analysed: A total of 584 patients attended the dental clinic during the recruitment period. Of these, 204 met the eligibility criteria, were enrolled in the study, completed the required study procedures, and were included in the final analyses.	4
		(b) Give reasons for non-participation at each stage: Patients not included in the final sample did not meet the eligibility criteria, met one or more exclusion criteria, or did not complete the required study procedures.	4
		(c) Consider use of a flow diagram: A flow diagram was not considered necessary because participant recruitment and inclusion criteria was not completed with following: 2 participants were <18 years old, 43 peticipants didn't have any natural teeth, and 335 participants didn't have detectable interdental clinical attachment loss.	-
Descriptive data	14*	(a) Give characteristics of study participants (eg demographic, clinical, social) and information on exposures and potential confounders: Demographic, anthropometric, behavioral, and clinical characteristics of the study participants are reported in the Results and participant characteristics table, including age, sex, BMI, smoking status, and periodontitis stage and grade.	7-8
		(b) Indicate number of participants with missing data for each variable of interest: No missing data were present for the variables included in the reported	6

		analyses of the final study sample (n = 204).	
Outcome data	15*	Report numbers of outcome events or summary measures: Summary measures for the main clinical periodontal outcomes (PPD, CAL, and BOP), as well as the distributions of CAL categories and periodontitis stage and grade, are reported in the Results section and corresponding tables.	7-16
Main results	16	(*a*) Give unadjusted estimates and, if applicable, confounder-adjusted estimates and their precision (eg, 95% confidence interval). Make clear which confounders were adjusted for and why they were included: Adjusted estimates from the multivariable regression models are reported as unstandardized regression coefficients (B), standardized coefficients (β), p-values, and 95% confidence intervals. Each model simultaneously included age, sex, BMI, smoking status, physical activity frequency, toothbrushing frequency, tooth mobility, education level, and the assessed dietary habits.	12-15
		(*b*) Report category boundaries when continuous variables were categorized: Category boundaries are explicitly reported for categorized continuous variables, including CAL categories and age groups (<35 and ≥35 years).	6-7,10-12,15-16
		(*c*) If relevant, consider translating estimates of relative risk into absolute risk for a meaningful time period: the study did not estimate relative risks	-
Other analyses	17	Report other analyses done—eg analyses of subgroups and interactions, and sensitivity analyses: Additional analyses included age-stratified analyses and analyses of periodontitis stage and grade according to age group.	15-16
**Discussion**			
Key results	18	Summarise key results with reference to study objectives: The Discussion summarizes the principal findings in relation to the study objective. In the fully adjusted analyses, selected dietary habits were independently associated with PPD and BOP, whereas no individual predictor was independently associated with CAL. BMI and physical activity were not independently associated with PPD, CAL, or BOP after simultaneous adjustment for the other variables included in the models.	16-19
Limitations	19	Discuss limitations of the study, taking into account sources of potential bias or imprecision. Discuss both direction and magnitude of any potential bias: The Discussion addresses the main limitations, including the cross-sectional design, potential recall and social desirability bias, use of a study-specific dietary questionnaire, single-center recruitment, limited generalizability, and the absence of an a priori sample size calculation.	19
Interpretation	20	Give a cautious overall interpretation of results considering objectives, limitations, multiplicity of analyses, results from similar studies, and other relevant evidence: The findings are interpreted in the context of the study objectives, methodological limitations, adjusted analyses, and previous evidence. Causal interpretations are avoided because of the cross-sectional study design.	16-20
Generalisability	21	Discuss the generalisability (external validity) of the study results: The Discussion addresses the external validity of the findings and acknowledges that recruitment from a single clinical setting in north-western Romania may limit generalizability to other populations and settings.	19
**Other information**			
Funding	22	Give the source of funding and the role of the funders for the present study and,	20

	if applicable, for the original study on which the present article is based: no external funding.

*Give information separately for exposed and unexposed groups.

Note: An Explanation and Elaboration article discusses each checklist item and gives methodological background and published examples of transparent reporting. The STROBE checklist is best used in conjunction with this article (freely available on the Web sites of PLoS Medicine at http://www.plosmedicine.org/, Annals of Internal Medicine at http://www.annals.org/, and Epidemiology at http://www.epidem.com/). Information on the STROBE Initiative is available at www.strobe-statement.org.
